# Electrospun Nanofiber Membranes from 1,8-Naphthimide-Based Polymer/Poly(vinyl alcohol) for pH Fluorescence Sensing

**DOI:** 10.3390/molecules27020520

**Published:** 2022-01-14

**Authors:** Le Xu, Xi Liu, Jiao Jia, Hao Wu, Juan Xie, Yongtang Jia

**Affiliations:** Guangdong-Hong Kong Joint Laboratory for New Textile Materials, Guangdong Functional Fiber and Textile Engineering Technology Research Center, School of Textile Materials and Engineering, Wuyi University, Jiangmen 529020, China; xlxule@163.com (L.X.); 17863625375@163.com (J.J.); wuhao_20211221@163.com (H.W.); anna_jxie@163.com (J.X.)

**Keywords:** electrospinning, nanofibers, fluorescence, sensors, pH

## Abstract

Accurately and sensitively sensing and monitoring the pH in the environment is a key fundamental issue for human health. Nanomaterial and nanotechnology combined with fluorescent materials can be emerged as excellent possible methods to develop high-performance sensing membranes and help monitor pH. Herein, a series of fluorescent nanofiber membranes (NFMs) containing poly-1,8-naphthimide derivative-3-[dimethyl-[2-(2-methylprop-2-enoyloxy)ethyl]azaniumyl]propane-1-sulfonate (PNI-SBMA) are fabricated by electrospinning the solution of PNI-SBMA blended with poly(vinyl alcohol) (PVA). The surfactant-like functionalities in side chains of PNI-SBMA endow the NFMs with outstanding hydrophilicity, and the naphthimide derivatives are sensitive to pH by photoinduced electron transfer effect, which contribute to highly efficient pH fluorescence sensing applications of NFMs. Specifically, the PNI-SBMA/PVA NFM with a ratio of 1:9 (NFM2) shows high sensitivity and good cyclability to pH. This work demonstrates an effective strategy to realize a fluorescent sensor NFM that has a fast and sensitive response to pH, which will benefit its application of pH sensor monitoring in the water treatment process.

## 1. Introduction

With the continuous acceleration of industrialization and urbanization, the subsequent water pollution issue has attracted considerable attention [1,2,3,4,5,6]. The pH value is an important indicator reflecting the basic properties of water. Moreover, the World Health Organization (WHO) has set the optimal pH range of drinking water to be 6.5–9.5 [7]. Importantly, long-term exposure to excessive acid or alkaline conditions can cause toxic effects on the skin, gastrointestinal tract, and nervous system [8,9,10,11]. Therefore, in tracking the level of disinfection and other water treatment processes, it is necessary to adjust the pH value through effective pH detection.

To date, a variety of pH sensing and measurement technologies have been developed, including luminescence imaging, fiber optic pH sensors, ion sensitive field effect transistors (ISFET), and various electrochemical methods [12,13,14,15,16]. Compared with other sensing methods, the optical sensing method shows higher sensitivity and selectivity of pH-based color and environmental pH [17,18]. For example, Chen et al. reported an 8-hydroxyquinoline-functionalized covalent organic framework (COF-HQ), which showed increasing fluorescent intensities against pH in the range from 1 to 5 [6]. Zhu et al. developed a multifunctional zwitterionic hydrogel that can simultaneously detect and sense pH and glucose levels [19].

Generally, compared with pH fluorescent sensing powders or hydrogels, pH fluorescent sensing nanomaterials have attracted much attention due to their advantages, such as an adjustable nanostructure, high porosity, and large specific surface area [20,21,22]. Meanwhile, pH fluorescence sensors based on nanomaterials including nanofibers, nanoparticles, carbon nanotubes, and quantum dots, etc., have made great progress recently [20,23,24,25]. Among these nanomaterials-based fluorescence sensors, nanofiber membranes (NFMs)-based fluorescence sensors show the characteristics of flexibility, low cost, and easy fabrication, which exhibits good potential for application [3,26]. Electrospinning is an effective method to obtain polymer-based NFMs, which has the characteristics of process flexibility, easy formation of pores, and high uniformity [17,27]. Thus, fluorescent NFMs fabricated by electrospinning display improved sensitivity and response rate [28,29,30,31,32]. Therefore, much attention has been devoted to fluorescent NFMs to explore their specific pH sensing properties. For example, Pan et al. integrated curcumin into fiber materials and prepared nanofiber dressings that can visually monitor the pH of wounds in situ in real time [33]. Kuo et al. prepared a fluorescence polymer and its electrospinning NFM for pH and Fe^3+^ fluorescence sensing application [34]. Therefore, the design and development of NFMs containing high-efficiency fluorescent polymers will benefit the realization of high-efficiency pH sensor monitoring in the water treatment process, such as pH detection of water before and after the multi-stage water-treatment separation membranes.

Naphthalimide-based structures exhibit excellent photophysical and fluorescence properties, which have been reported and used as pH sensors in solution states [35,36,37]. However, there are few research reports on naphthalimide-based fluorescent nanofiber membranes. Herein, we report a series of NFMs containing poly-1,8-naphthimide derivative-zwitterionic copolymer (PNI-SBMA) fabricated by electrospinning the solution of PNI-SBMA blended with poly(vinyl alcohol) (PVA) for pH fluorescence sensing. The relationship between the nanofibrous structures and pH fluorescence sensing performance of NFMs has been systematically studied. This work provides a simple strategy to prepare pH fluorescent sensing NFMs, which will benefit its application of pH sensor monitoring in the water treatment process.

## 2. Materials and Methods

### 2.1. Materials

4-Bromo-1,8-naphthalic anhydride, 1-methylpiperazine, ethanolamine, methacryloyl chloride, 2-(dimethylamino)ethyl methacrylate, 2,2′-azobis(2-methylpropionitrile) (AIBN), 1-methyl-2-pyrrolidinone (NMP), 1,3-propane sultone, and organic solvents were purchased from Energy Chemicals Co., Ltd. (Shanghai, China). PVA 1788 (alcoholysis degree: 87.0–89.0% (mol/mol), Mn = 80 kDa) was purchased from Aladdin Reagents Co., Ltd. (Shanghai, China). All chemicals and solvents were of analytical grade, and no further purification was required.

### 2.2. Methods

#### 2.2.1. Synthesis of PNI-SBMA

**Scheme 1:**
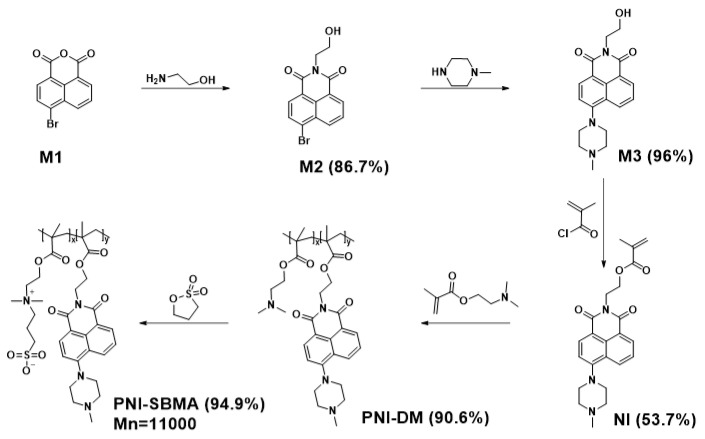
The synthetic route of NI and PNI-SBMA.

The synthetic route of PNI-SBMA is shown in Scheme 1. NI was synthesized according to the method reported in the literature [14] In the reaction tube, 2-(dimethylamino)ethyl methacrylate (1 g, 2.5 mmol), NI (0.39 g, 2.5 mmol), and AIBN (0.017 g, 0.104 mmol) were dissolved in NMP (1.8 mL). The reaction tube was ventilated with N_2_ four times at −20 °C to remove O_2_. Thereafter, the mixture was heated to 70 °C and continuously stirred for 48 h. Afterward, the solution was cooled to room temperature, and a minute amount of dichloromethane was added and precipitated in petroleum ether/ethyl acetate (*v/v* = 3:2) to remove unreacted monomers. The mixture was filtered and vacuum dried at 45 °C to obtain the polymer substrate (PNI-DM: 1.26 g, yield 90.6%). Under a N_2_ atmosphere, PNI-DM (1 g) was completely dissolved in tetrahydrofuran (60 mL), and excess 1,3-propane sultone was added to the flask in batches. After refluxing for 72 h, the solvent was removed in vacuo, and the product was completely dissolved in 5 mL of deionized water at 50 °C. Precipitation was performed in anhydrous methanol to remove unreacted raw materials. The mixture was filtered and vacuum dried at 50 °C to obtain the poly-1,8-naphthimide derivative-zwitterionic (PNI-SBMA: 1.5 g, yield 94.9%).

#### 2.2.2. Preparation of Electrospun NFMs

The electrospinning process was performed on an E05 electrospinning apparatus (supplied by Lepton Technology Co., Ltd., Foshan, China). Firstly, different ratios of PNI-SBMA/PVA (1:14, 1:9, and 1:4 in wt./wt., offering the corresponding nanofiber membranes named as NFM1, NFM2, and NFM3, respectively) were dissolved into deionized water, offering the corresponding electrospinning solutions with a concentration of 10 wt.%. Then, the nanofibers were collected by a rotating metal cylinder (100 r/min) covered with release paper, with an immobile distance of 12 cm away from the needle tip of the spinneret (0.7 mm inner diameter). The spinning voltage and fluid flow rate were set to 20 kV and 1 mL/h, respectively. Finally, the obtained nanofiber membranes were cross-linked with glutaraldehyde and dried in a vacuum oven at 70 °C for another 12 h.

#### 2.2.3. Characterization

The NMR spectra were recorded on a Bruker-500 MHz spectrometer at room temperature. The deuterated solvents of NI, PNI-DM, and PNI-SBMA were CDCl_3_, CDCl_3_, and D_2_O, respectively. FTIR spectra data were collected with a Nicolet iS50 Fourier transform infrared spectrometer in the 400–4000 cm^−1^ region. The solid powder samples of PNI-DM and PNI-SBMA (1 mg) were taken, respectively, and the KBr tableting method was used to make transparent flakes, in which the weight ratio of KBr to the sample is 100:1. The molecular weight of PNI-SBMA was determined by a Waters GPC 2410 in DMF using a calibration curve with polystyrene standards. Scanning electron microscopy (SEM) characterization and energy dispersive spectroscopy (EDS) mapping were conducted using a ZEISS Sigma500 instrument. The corresponding nanofiber membranes were prepared with a size of 1 cm × 1 cm, and sprayed with platinum on the surface for SEM measurements. Water contact angles of the NFMs were recorded with a contact angle tester (DSA100-Kruss, FSP GROUP, Hamburg, Germany). A 2 μL deionized water droplet was droplet on the surface of the membrane for measurement. Each membrane was tested five times for statistics. The nanofiber membranes were immersed in solutions of different pH values for 30 min, after which fluorescence optical microscope images of NFMS were taken by the inverted fluorescence microscope (Axio Observer A1, ZEISS, Oberkochen, Germany). The photophysical properties were studied by fluorescence spectroscopy, and the experimental data were recorded using a fluorescence spectrophotometer (RF-6000, SHIMADZU, Kyoto, Japan). The NFM was fixed in a cuvette and filled with alkaline aqueous solutions. Each measurement is maintained for 15 min to ensure that the chelation reaction reaches equilibrium.

## 3. Results and Discussion

### 3.1. Design, Synthesis, and Characterization of PNI-SBMA

**Figure 1:**
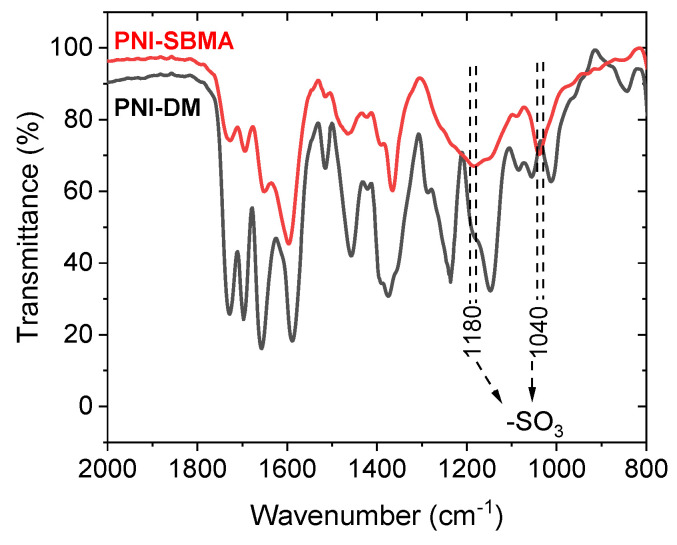
Fourier transform infrared (FTIR) spectrum of the PNI-DM and PNI-SBMA.

The chemical structure of the poly-1,8-naphthimide derivative-zwitterionic copolymer (PNI-SBMA) is shown in Scheme 1. The naphthalimide linked with piperazine unit ensures the specific fluorescent characteristics, including high extinction coefficient, excellent quantum yield, good light stability, and relatively long emission wavelength [38,39]. Meanwhile, the piperazine unit can be protonated and deprotonated under acidic and alkaline conditions, offering different intermolecular photoinduced electron transfer levels and fluorescence intensities, which ensures the pH fluorescence sensing property of the target polymer [40]. The polymer PNI-SBMA contains a soft main chain of polymethacrylic derivatives to ensure spinnability when blended with PVA, while 1,8-naphthimide- and zwitterionic-based side chains were anticipated to offer pH fluorescence sensing and hydrophilic properties, respectively. The synthetic route of PNI-SBMA is shown in Scheme 1, and its detailed procedure is described in the Materials and Methods part. PNI-SBMA was prepared by two steps: (1) the precursor polymer (PNI-DM) was synthesized by free-radical polymerization from 1,8-naphthimide derivative (N1) and 2-(dimethylamino)ethyl methacrylate and (2) the target polymer obtained from post-treatment of PNI-DM with 1,3-propane sultone. The resulting product can be readily dissolved at room temperature in water. The chemical structures of the monomer and polymers were preliminarily characterized by ^1^H NMR measurements (Figure S1, see Supplementary Materials) [37]. Fourier-transform infrared (FTIR) spectroscopies of PNI-DM and PNI-SBMA (Figure 1) were performed to further confirm their chemical structures. Compared to the FTIR result of PNI-DM, two new bands of PNI-SBMA at 1180 and 1040 cm^−1^ were caused by the stretching vibrations of the SO_3_^−^ group, suggesting the successful preparation of the target polymer [41]. The number-average molecular weight (Mn) and weight-average molecular weight (Mw) of PNI-SBMA were estimated by gel permeation chromatography (GPC) using polystyrene as the standard and DMF as the eluent. As shown in Figure S2d,e and Table S1, the Mn and Mw of PNI-SBMA are ~11 kDa and ~23 kDa, respectively, offering its polydispersity index (PDI = Mw/Mn) of 2.19. These results indicate that the target polymer was successfully synthesized through our design strategy.

### 3.2. Preparation and Characterization of NFMs

**Scheme 2:**
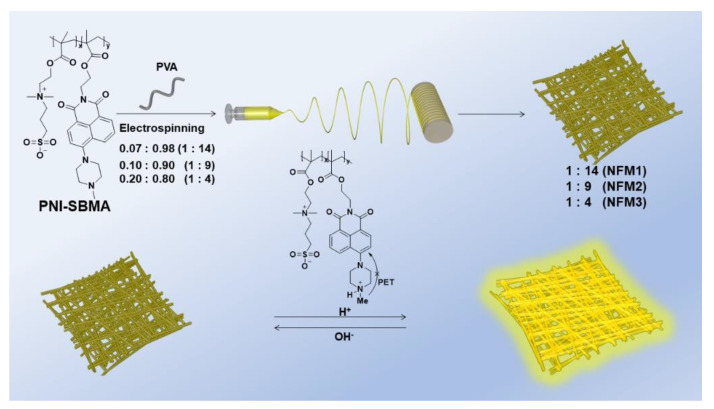
Schematic diagram of the fabrication process of NFMs and the fluorescence sensing for pH.

**Figure 2:**
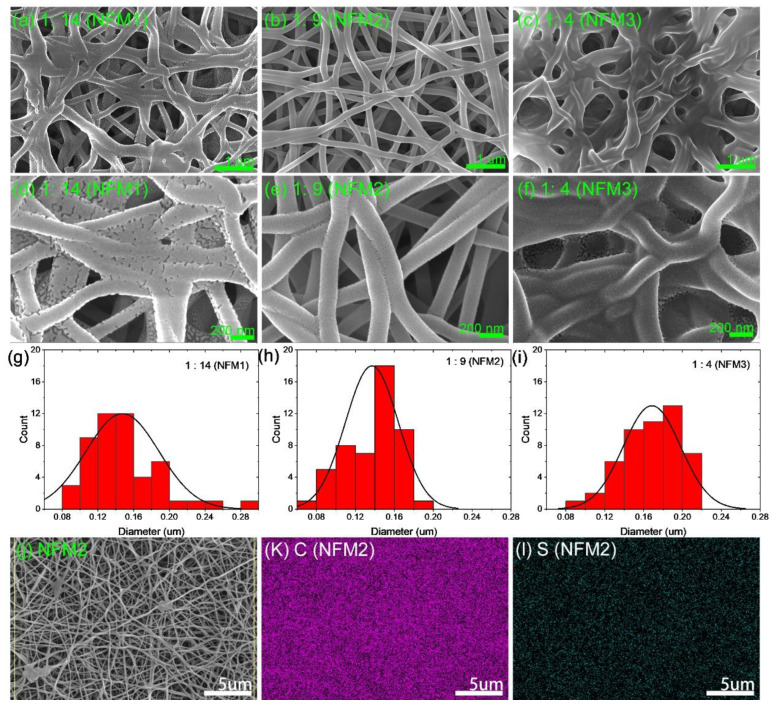
Scanning electron microscopy (SEM) images of the poly-1,8-naphthimide derivative-zwitterionic/poly(vinyl alcohol) (PNI-SBMA/PVA) NFMs and diameter distribution at different ratios: 1:14 (**a**,**d**,**g**), 1:9 (**b**,**e**,**h**), and 1:4 (**c**,**f**,**i**). (**j**) SEM image of NFM2 with the EDS mapping spectrum of the (**k**) C and (**l**) S element.

The detailed preparation of PNI-SBMA@PVA NFMs by electrospinning process is described in the Materials and Methods section. As shown in Scheme 2, the weight ratio of PNI-SBMA to PVA is set to be 1:14, 1:9, and 1:4, leading to the corresponding membranes with names of NFM1, NFM2, and NFM3, respectively. The morphologies of the three nanofiber membranes were tested using scanning electron microscopy (SEM). The corresponding SEM images are shown in Figure 2a–f. Generally, to avoid the dissolution of pristine PVA-based nanofiber membranes, the crosslinked structures of NFMs were obtained by post-treatment of the NFMs through acetal reaction of PVA and glutaraldehyde. Correspondingly, as shown in Figure 2a–f, the NFMs show micro-nano scale entangled structures with abundant nodes dispersed in their nanofibrous networks. Visually, NFM2 with a moderate PNI-SBMA/PVA (1:9) ratio shows a nanofiber structure with clear boundaries in its 3D network, while it displays a spider net ambiguous morphology in the other two ratios. The related spider net ambiguous morphologies may be attributed to the instabilities polymer stream of straight jet or after the Taylor cone, which is similar to the reported results [42,43,44,45]. To further confirm these intuitive nanofiber morphologies, we measured the diameter distribution of the corresponding nanofibers. As shown in Figure 2g–i, the average diameters of NFM1, NFM2, and NFM3 are 147.4 ± 40.5, 136.9 ± 26.9, and 168.6 ± 29.2 nm, respectively, suggesting a slightly smaller average diameter, and a more uniform diameter distribution of NFM2 than other NFMs. Moreover, energy dispersive spectroscopy (EDS) mapping of NFMS was performed and shown in Figure 2j–l and Figure S3, displaying that the C, S, N, and O element (denoted as PNI-SBMA) is uniformly distributed on the surface of NFM2, which confirms its uniform structure. Thus, the subsequent pH fluorescence sensing measurement was preferably performed based on NFM2.

**Figure 3:**
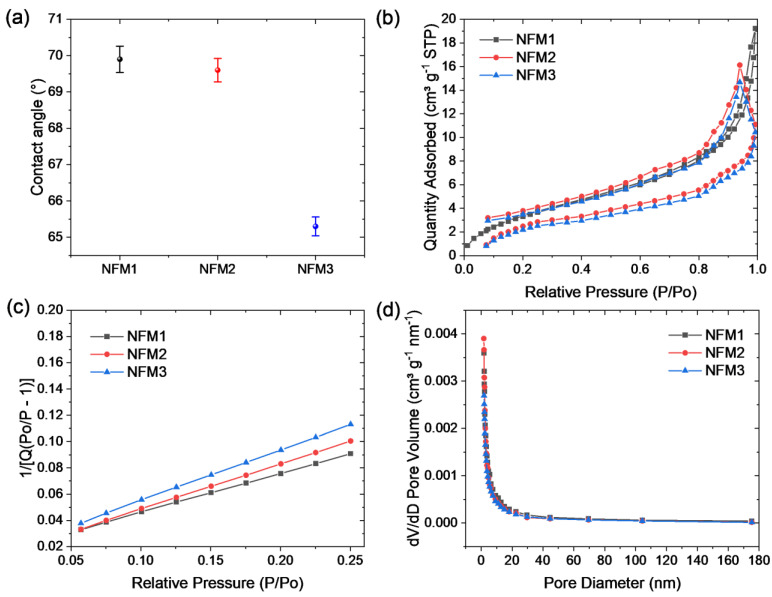
(**a**) Average water contact angles of PNI-SBMA/PVA NFMs in different proportions, (**b**) nitrogen adsorption isotherms of PNI-SBMA/PVA NFMs, (**c**) the linear plots of the corresponding Brunauer-Emmett-Teller (BET) surface areas of PNI-SBMA/PVA NFMs, and (**d**) pore size distributions of PNI-SBMA/PVA NFMs.

Contact angle measurements were performed to examine the hydrophilicity of the membranes. As shown in Figure 3a and Figure S5, the average water contact angles of NFM1, NFM2, and NFM3 are 69.9° ± 0.36°, 69.6° ± 0.32°, and 65.3° ± 0.26°, respectively, which demonstrated that the NFMs with PNI-SBMA contents offer good hydrophilicity properties. Therefore, the prepared NFM can fully and evenly contact the solution, ensuring a good fluorescence sensing test. In addition, to quantitative evaluate and compare the surface areas and pore sizes of NFMs, we then measured the relative nitrogen adsorption experiments. Nitrogen adsorption isotherms (Figure 3b,c) exhibited Brunauer–Emmett–Teller (BET) surface areas of 8.1, 7.5, and 7.8 m^2^ g^−1^ for NFM1, NFM2, and NFM3, respectively. Correspondingly, as shown in Figure 3d, NFM1, NFM2, and NFM3 exhibit an average pore diameter of 12.3, 8.5, and 10.5 nm, respectively. The smallest average pore diameter of NFM2, compared to NFM1 and NFM3, verifying the fine micro-nano scale morphology of NF2, which are shown in the SEM images. Overall, the result of the surface areas and average pore diameters of the three nanofiber membranes is consistent with the morphologies discussed above, suggesting NFM2 preferably act as the membrane for the subsequent pH fluorescence sensing measurement.

### 3.3. pH Fluorescence Sensing Properties

**Figure 4:**
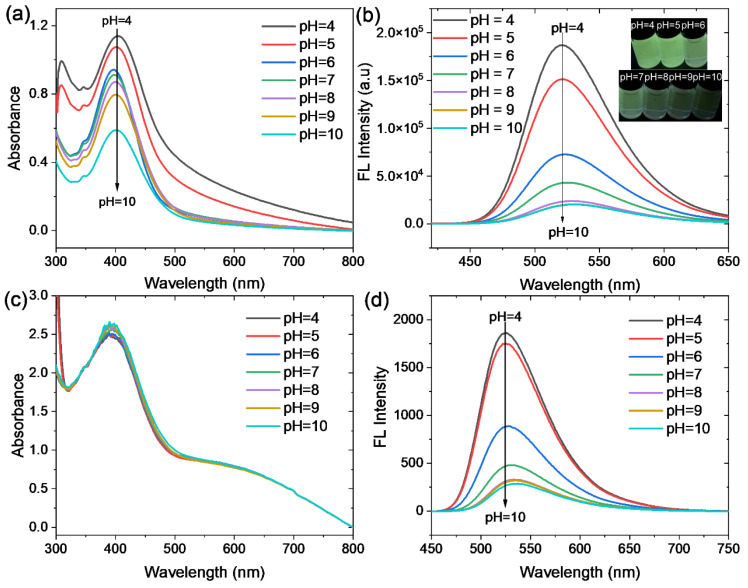
The UV absorption spectra of (**a**) PNI-SBMA and (**c**) PNI-SBMA/PVANFM in solution with pH = 4–10; the fluorescence spectra of (**b**) PNI-SBMA and (**d**) PNI-SBMA/PVA NFM in solution with pH = 4–10.

**Figure 5:**
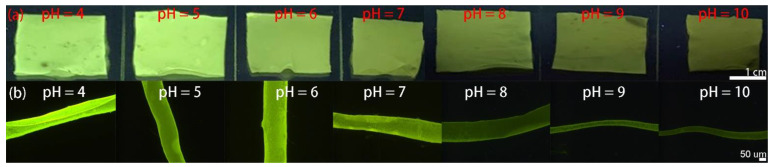
(**a**) The surface luminescence and (**b**) single nanofiber-inverted fluorescence microscope images of NFM2 at different pH levels.

To evaluate the pH fluorescence sensing properties of NFM2, the ultraviolet-visible (UV-vis) absorption and fluorescence spectra of the corresponding polymer and membrane under varied pH solutions were performed. Figure 4a shows the UV absorption spectra of PNI-SBMA solutions at different pH values. With the pH value in the range of 4 to 10, PNI-SBMA solutions all exhibit absorption peaks at 402 nm, while the absorption intensities gradually decrease along with the pH value increased from 4 to 10. Correspondingly, Figure 4b displays the fluorescence spectra of PNI-SBMA solutions at different pH values. It is clearly shown that the fluorescence intensities of the fluorescence peak at 524 nm of PNI-SBMA solutions decrease as the pH value increases from 4 to 10. The illustration of Figure 4b clearly shows this phenomenon, which demonstrated that the pH fluorescence sensing property of pure BPI-SBMA solution. The pH fluorescence sensing property of PNI-SBMA can be attributed to the protonation of the piperazine group weakens the positron emission tomography between the lone electron pair of the -NCH_3_ group and the naphthimide fluorophore (Figure S1) [46]. When PNI-SBMA is exposed to an acidic environment, the piperazine group is protonated, the photoinduced electron transfer between the naphthimide fluorophore and the piperazine group is inhibited, and the fluorophore emits strong fluorescence [46]. Conversely, when PNI-SBMA is exposed to an alkaline environment, the photoinduced electron transfer between the naphthimide fluorophore and the piperazine group is enhanced, and the fluorescence of the fluorophore is further quenched [46,47,48]. Considering the excellent pH fluorescence sensing property of the PNI-SBMA solution, as well as the good nanofiber morphology of NFM2, we then focus on the fluorescence sensing property of NFM2 under varied pH conditions. Figure 4c displays the absorption curves of NFM2 immersed in aqueous with the pH value in the range of 4 to 10, showing similar absorption spectra with long trails, which may be attributed to the dispersion of PNI-SBMA in the nanofibrous structure of NFM2. However, similar to the fluorescence measurements of PNI-SBMA solutions under varied pH, NFM2 exhibits obvious pH fluorescence sensing property when immersed in aqueous solution with a pH value in the range of 4 to 10 (Figure 4d). Figure 4d clearly shows that the fluorescence intensity of NFM2 decreased as the pH increased from 4 to 10. Furthermore, Figure 5 shows the surface luminescence and single nanofiber-inverted fluorescence microscope images of NFM2 at different pH levels. As shown in Figure 5a, the membranes display an obvious decrease in brightness of surface luminescence as the pH increased from 4 to 10. Meanwhile, a similar phenomenon on pH fluorescence sensing of a single nanofiber of NFM2 was verified in Figure 5b. In a word, NFM2 shows enhanced fluorescence luminescence intensity under acid condition (pH = 4–6) and weakened fluorescence luminescence intensity under base condition (pH = 8–10). These results demonstrated that a PNI-SBMA-based nanofiber membrane offers good pH fluorescence sensing performance, which may benefit from other application, such as a food preservation label.

**Figure 6:**
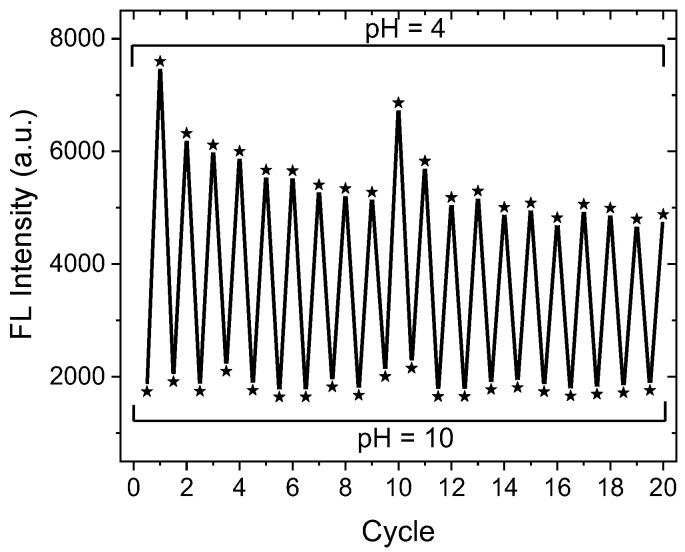
Circulation diagram of NFM2 in pH (4 and 10) solutions.

Since the stability is an important factor for the future pH fluorescence sensing application of the membranes, photobleaching and cycling fluorescence measurements were used to investigate the stability of NFM2. As shown in Figure S4, after continuous 24 h of strong light from the xenon arc lamp, the fluorescence intensity of NFM2 was reduced by about 32%, suggesting its qualified light stability. Moreover, Figure 6 shows the reversibility test of NFM2 in aqueous solution with a pH value of 4 and 10, while the changes of its fluorescence intensities (enhanced in acid solution and weaken in base solution) still remain stable after 20 cycles. Overall, these results demonstrated that the NFM2 offers stable pH fluorescence sensing property.

## 4. Conclusions

In conclusion, we have developed a series of fluorescent NFMs containing poly-1,8-naphthimide derivative-zwitterionic copolymer (PNI-SBMA), which are fabricated by electrospinning the solution of PNI-SBMA blended with PVA. Specifically, PNI-SBMA is uniformly dispersed in the nanofiber membrane, and NFM2 shows a good porous micro-nano morphology structure. More importantly, NFM2 shows good pH fluorescence sensing performance with high sensitivity to pH (4–10), and exhibits stable pH fluorescence sensing property. This work demonstrates an effective strategy to achieve fluorescent NFMs and has potential applications in pH sensor monitoring in the water treatment process.

## Data Availability

Not applicable.

## Supplementary Materials

The following are available online, Figure S1: PNI-SBMA sensing mechanism, Figure S2: ^1^H NMR of the materials and GPC of PNI-SBMA, Figure S3: EDS mapping spectrum of N and O elements, Figure S4: The photobleaching property of the fibers with xenon arc lamp, Figure S5: Water contact angle images, Figure S6: Fluorescence spectra of PNI-SBMA/PVA nanofiber membranes in different ratios, Table S1: Molecular weights and molecular weight distribution of PNI-SBMA.

## Sample Availability

Samples of the compounds PNI-DM and PNI-SBMA are available from the authors with appropriate requests.
