# Supplementary material for: Electrospun Nanofiber Membranes from 1,8-Naphthimide-Based Polymer/Poly(vinyl alcohol) for pH Fluorescence Sensing
Source: Molecules. 2022 Jan 14;27(2):520. doi: 10.3390/molecules27020520 (PMC8778194; doi:10.3390/molecules27020520)
Supplement: Supplementary file 1 [file molecules-27-00520-s001.zip › molecules-1539855-supplementary.pdf]

Supplementary Materials

Electrospun Nanofiber Membranes from 1,8-Naphthimide-Based Polymer/Poly(Vinyl Alcohol) for pH Fluorescence Sensing

Le Xu[1], Xi Liu[1,*], Jiao Jia[1], Hao Wu[1], Juan Xie[1], Yongtang Jia[1,*]

[1] School of Textile Materials and Engineering, Guangdong Functional Fiber and Textile Engineering Technology Research Center, Guangdong-Hong Kong Joint Laboratory for New Textile Materials, Wuyi University, Jiangmen 529020, China

* Correspondence: liuxi_wyu@163.com; yongtjh@163.com

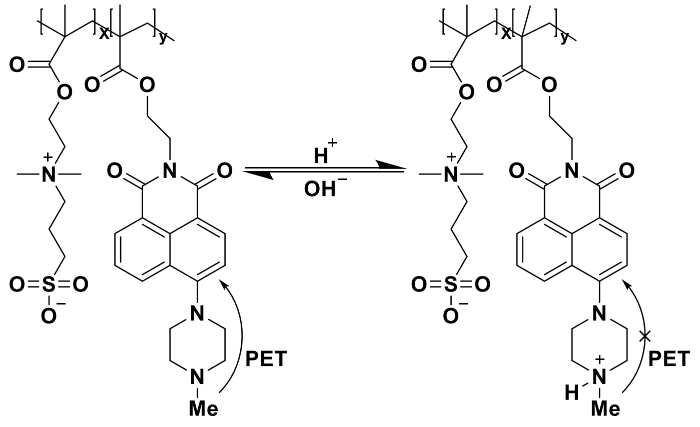

Figure S1. PNI-SBMA sensing mechanism for pH.

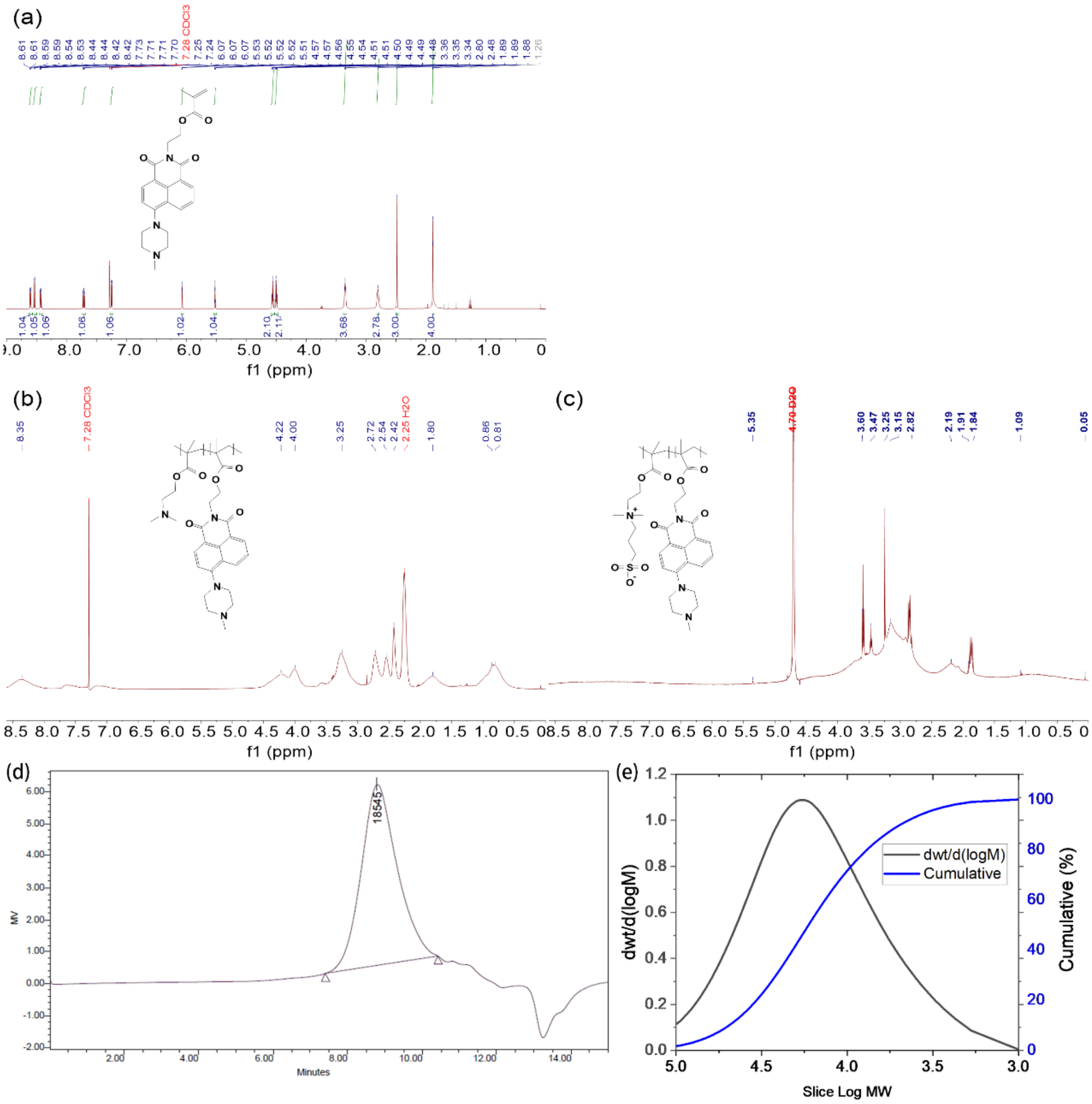

Figure S2. ^{1}H NMR of NI (a), PNI-DM (b), and PNI-SBMA (c). The relative GPC chromatogram (d) and calibration chart (e) of PNI-SBMA.

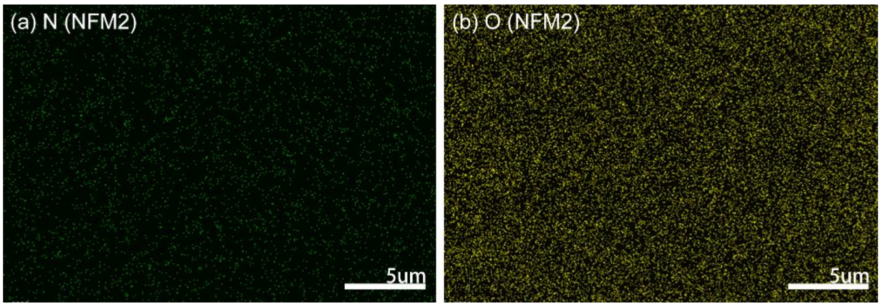

Figure S3. The EDS mapping spectrum of the (a) N and (b) O element.

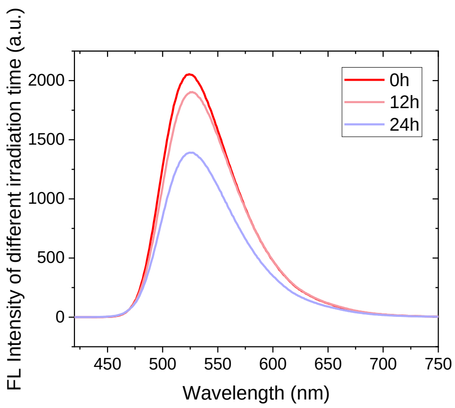

Figure S4. The photobleaching property of the fibers with xenon arc lamp.

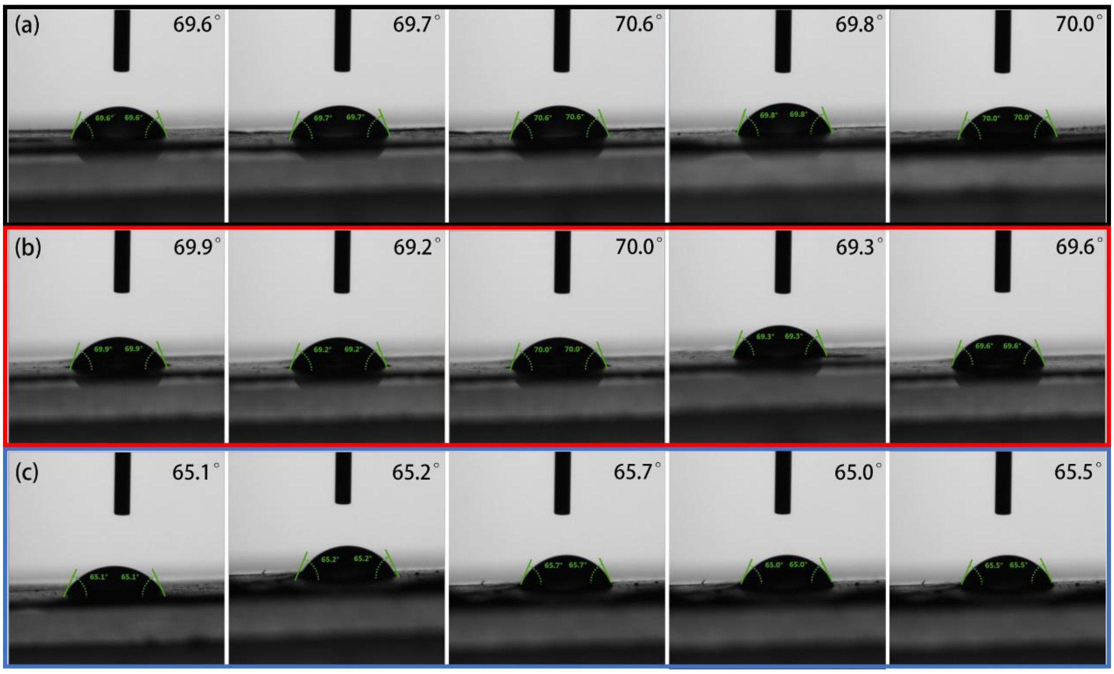

Figure S5. Water contact angle images of PNI-SBMA/PVA nanofiber membranes with different ratios (a) 1:14, (b) 1:9, (c) 1:4.

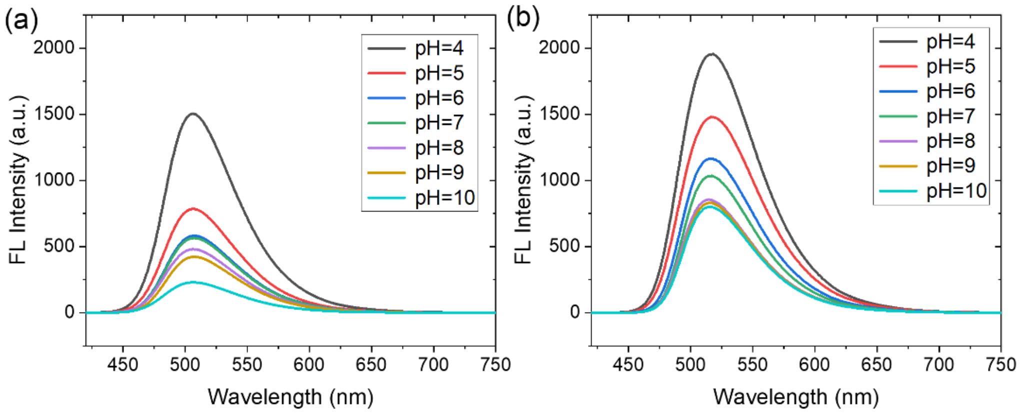

Figure S6. Fluorescence spectra of PNI-SBMA/PVA nanofiber membranes in different ratios (a) 1:14 (NFM1) and (b) 1:4 (NFM3) in a solution of pH = 4 – 10. It should be noted that, compared to NFM2, NFM1 and NFM3 also show similar fluorescence properties under different pH solutions.

Table S1. Molecular weights and molecular weight distribution of PNI-SBMA determined via GPC.

Sample	Mn	Mw	PDI (Mw/Mn)
PNI-SBMA	10539	23131	2.19
